# RETRACTED: Khan et al. Norfloxacin Loaded Lipid Polymer Hybrid Nanoparticles for Oral Administration: Fabrication, Characterization, In Silico Modelling and Toxicity Evaluation. *Pharmaceutics* 2021, *13*, 1632

**DOI:** 10.3390/pharmaceutics17121624

**Published:** 2025-12-18

**Authors:** Muhammad Asghar Khan, Shahzeb Khan, Mohsin Kazi, Sultan M. Alshehri, Muhammad Shahid, Shafi Ullah Khan, Zahid Hussain, Muhammad Sohail, Muhammad Shafique, Hajra Afeera Hamid, Mahwish Kamran, Abdelbary Elhissi, Muhammad Wasim, Hnin Ei Thu

**Affiliations:** 1 Department of Pharmacy, University of Malakand, Chakdara 18800, Pakistan; asghar247@yahoo.com (M.A.K.); afeera85@gmail.com (H.A.H.); arianmahwish@gmail.com (M.K.); 2 Discipline of Pharmaceutical Sciences, School of Health Sciences, University of Kawazulu Natal, Durban X54001, South Africa; 3 Department of Pharmaceutics, College of Pharmacy, King Saud University, Riyadh 11451, Saudi Arabia; mkazi@ksu.edu.sa (M.K.); salshehri1@ksu.edu.sa (S.M.A.); 4 Department of Pharmacy, Sarhad University of Science and Information Technology, Peshawar 25000, Pakistan; shahidsalim_2002@hotmail.com; 5 Department of Pharmacy, Abasyn University Peshawar, Peshawar 25000, Pakistan; shafiullahpharmd@gmail.com; 6 Department of Pharmaceutics & Pharmaceutical Technology, College of Pharmacy, University of Sharjah, Sharjah 27272, United Arab Emirates; zhussain@sharjah.ac.ae; 7 Research Institute for Medical and Health Sciences (SIMHR), University of Sharjah, Sharjah 27272, United Arab Emirates; 8 Department of Pharmacy, COMSATS University Abbottabad Campus, Abbottabad 45550, Pakistan; Msmarwat@cuiatd.edu.pk (M.S.); wassypharmacist@gmail.com (M.W.); 9 Department of Pharmaceutical Sciences, College of Pharmacy-Boys, Al-Dawadmi Campus, Shaqra University, Shaqra, Riyadh 11451, Saudi Arabia; shafiqueph@yahoo.com; 10 College of Pharmacy, QU Health and Office of VP for Research and Graduate Studies, Qatar University, Doha 2713, Qatar; aelhissi@qu.edu.qa; 11 Research and Innovation Department, Lincolon University College, Petaling Jaya 47301, Malaysia; eithuu287@gmail.com; 12 Innoscience Research Institute, Subang Jaya 47650, Malaysia

The journal retracts the article “Norfloxacin Loaded Lipid Polymer Hybrid Nanoparticles for Oral Administration: Fabrication, Characterization, In Silico Modelling and Toxicity Evaluation” [1] cited above.

Following publication, concerns were brought to the attention of the Editorial Office regarding the integrity of a figure presented in this publication [1].

Adhering to our standard procedure, an investigation was conducted by the Editorial Office and Editorial Board that identified indications of inappropriate editing and partial duplication between Figure 4 in [1] and Figure 8 in [2], presenting different experimental conditions. While the authors collaborated with this process, raw material meeting the journal’s requirements for original images could not be provided for evaluation by the Editorial Board. Consequently, the Editorial Board has lost confidence in the reliability of the findings and has decided to retract this publication, as per MDPI’s retraction policy (https://www.mdpi.com/ethics#_bookmark30).

This retraction was approved by the Editor-in-Chief of the journal *Pharmaceutics*.

The authors did not agree to this retraction.
